# Spread and Endemicity of Cholera in India: Factors Beyond the Numbers

**DOI:** 10.1093/infdis/jiab436

**Published:** 2021-09-22

**Authors:** Gautam Kumar Saha, Nirmal Kumar Ganguly

**Affiliations:** 1 Apollo Hospitals Educational and Research Foundation, Delhi, India; 2 Former DG, Indian Council of Medical Research, Delhi, India

**Keywords:** cholera, hotspots, surveillance

## Abstract

Cholera outbreaks currently account for 1.3 to 4.0 million cases and cause between 21 000 and 143 000 deaths worldwide. Cholera is preventable by proper sanitization and immunization; however, in many developing nations such as India, cholera disease is endemic.

The surveillance system in India does not adequately capture the actual number of cases. As a result, it is important to utilize limited public health resources correctly in India and other developing counties more effectively to reach vulnerable communities. In this study, we analyze how studies make sense of cholera transmission and spread in India from 1996 to 2015. Furthermore, we analyze how a more sensitive surveillance system can contribute to cholera eradication by giving rise to outbreak preparedness.

In 2021, the World Health Organization (WHO) reported that there will be 1.3 to 4 million cases of cholera. Cases were reported in all continents: 17 countries in Africa, 12 in Asia, 4 in Europe, and 4 in the Americas; the United States and Oceania also reported cholera cases. Haiti, the Democratic Republic of the Congo, Vietnam, Yemen, Peru, Somalia, and Tanzania accounted for 80% of all cases [1, 2].

The WHO/UNICEF report that basic infrastructure facilities are lacking for approximately 700 million people and that cholera and enteric diseases are endemic and spreading [3]. In the 1990s, in Peru alone, cholera claimed over 10 000 lives. Several outbreaks took place in Southern and Central Africa in the 1990s and through the 2000s [4].

After the 2010 earthquake in Haiti and the Dominican Republic, a breakdown in basic water, sanitation and hygiene (WASH) infrastructure occurred. The abrupt absence of functional sanitation systems and lack of potable drinking water was a major factor leading to an outbreak of cholera. Climatic conditions also contributed to an increase in disease spread. In the aftermath of Hurricane Matthew, an upsurge of cholera cases took place in Haiti [5, 6]. In 2017–2018, Yemen faced the largest outbreak of cholera in its history, and this was complicated by civil unrest. The WHO estimated that more than 1 million people were infected due to cholera and there were more than 2000 deaths [7].

*Vibrio cholerae* O1 and O139 strains cause cholera disease. In persons with cholera, severe cases of diarrhea are also caused by the cholera toxin [8].

Cholera is an acute, diarrheal illness caused by infection of the intestine with the toxigenic bacterium *V cholerae*. The infection is often mild or without symptoms, but it can be severe. This toxin-mediated disease can rapidly lead to severe dehydration and death if left untreated. In serious cases of cholera, delayed treatment can be fatal. In low-income countries and conflict zones, the health system may be nonexistent [7, 8]. Hence, the prevention of the disease is of paramount importance.

## REASONS FOR CHOLERA SPREAD

UNICEF reports that the sanitation goals in the Millennium Development Program have not been met. For millions of people, clean drinking water, basic sanitation facilities, and sewage treatment systems are not present. The majority of such communities are found in rural areas that are devoid of the means to obtain and sustain better sanitation. An estimated 700 children are losing their lives every day because of diarrhea [9].

Cholera is frequently spread by the fecal-oral route. Untreated human excreta are a major cause of cholera. UNICEF estimates that half of the world’s human excreta remains untreated by sewage systems. Open defecation is still practiced globally by approximately 673 million people. Among school-age children, an estimated 373 million, do not have adequate sanitation facilities at home or in schools [9].

In 2014, the government of India started the Swachh Bharat Mission to provide rural and urban poor communities with subsidies for the construction of toilets [10]. However, not all communities have benefited from the scheme. At one time, almost half of the population in India defecated in the open. In 2014, WHO/UNICEF estimated that India accounted for 60% of the global population engaging in open defecation [11]. UNICEF reported that universal sanitation programs have not reached all populations [12]. Cholera vaccine immunization for populations in endemic areas may be the best and most equitable method for prevention.

## VACCINATION TO ERADICATE CHOLERA DISEASE AND ITS SPREAD: RECENT HISTORY, NEW INITIATIVES, AND WHO-PREQUALIFIED PRODUCTS

Cholera vaccines provide short-term protection; from 3 to 5 years. Vaccines that have been prequalified by the WHO are available. In cholera-endemic areas, if vaccination can be implemented, then disease spread can be controlled. Vietnam has suffered cholera outbreaks since 1964. Based upon the results of a clinical trial of a Swedish vaccine in Bangladesh, the Vietnamese government produced a vaccine containing killed whole-cell *V cholera*. The vaccine was different from the Swedish vaccine because it lacked the cholera toxin B subunit and contained the O139 strain of *V cholera* to the vaccine. This was done when it was found that outbreaks in India and Bangladesh contained O139. Clinical trials of Vietnam’s oral cholera vaccine (OCV) were demonstrated to be safe and 66% efficacious [13, 14]. The bivalent oral vaccine was found to be safe and efficacious, and it was locally produced in Vietnam by the Company for Vaccine and Biological Production No. 1 (VABIOTECH).

Vietnam was the first country to incorporate the cholera vaccination for cholera in its immunization program in the 1990s. The incidence of cholera was markedly reduced in the country after 1997 [14].

After the success of the VABIOTECH vaccine, the International Vaccine Institute (IVI), based in South Korea, developed a new formulation for the vaccine, which received WHO prequalification, and the IVI licensed it around the world. The Indian manufacturer Shantha Biotech licensed the oral cholera vaccine version from IVI. The vaccine has an efficacy rate of over 65% and protection lasts for up to 5 years. The price in Indian Rupees (INR) is 120 (less than US $2) per dose. The vaccine is known by the brand name Shancaol. This is particularly useful for infants because it does need to have a buffer for delivery. Shantha Biotech is a subsidiary of Sanofi-Aventis (France), and the vaccine is manufactured under its brand in India [15].

After the vaccine was licensed for production in India, a feasibility study was performed to demonstrate the effectiveness in an endemic area. The Odisha region was chosen; it has a low socioeconomic development index. The 2-year study demonstrated that in this cholera-endemic area, after vaccination, there was a 69% decrease in new cases. Thus, this provides strong evidence that a vaccine alone can prevent cholera and that a vaccination program can be sustained by the Public Health System.

The International Vaccine Institute, Korea concluded that more than 1 manufacturer was needed to meet the global cholera vaccine demand. In 2010, EuBiologics, a Korean company, became the second manufacturer of the IVI cholera vaccine. Their vaccine was prequalified by the WHO as well in 2015 and branded as Euvichol. EuBiologics scaled up production capabilities and manufactured 25 million doses in 2018, with the potential to increase capacity as needed for global vaccine stockpiles [16]. The International Vaccine Institute also transferred the technology to Incepta Vaccine Ltd. in Bangladesh to produce a vaccine called Cholvax. This vaccine is produced using Good Manufacturing Practices (GMP) and WHO guidelines. The vaccine has undergone clinical trials that demonstrate its bioequivalence to Shanchol [17]. Cholvax was introduced into Bangladesh in 2020 in large- scale immunization campaigns. Oral cholera vaccines began in the country, and a national immunization program is in place aimed at eliminating the disease by 2030 [18].

Dukoral is another vaccine that is prequalified by WHO and manufactured by Valneva in Sweden. The vaccine contains nonlive, whole-cell *V cholera* with a recombinant fragment (B subunit) of the cholera toxin. The vaccine also gives short-term protection against the enterotoxigenic *Escherichia coli* strain that makes heat-labile and heat-stable toxins. It is more expensive than the vaccines manufactured in India or Korean and priced at INR 320 (US $4.50) per dose. Dukoral was shown to be safe and efficacious in several trials in Bangladesh and Peru.

Dukoral requires 2 doses, which are administered with a buffer solution. After the first dose, it takes approximately 3 weeks to develop protection against cholera infection. After the second dose, the protection is greatest in the first 6 months. Dukoral provides immunity that lasts up to 3 years.

The US Food and Drug Administration recently approved a single-dose, live oral cholera vaccine, Vaxchora (lyophilized CVD 103-HgR). The Advisory Committee on Immunization Practices voted to approve the vaccine for adults 18 to 64 years old who are traveling to an area of active cholera transmission [19].

Under WHO guidance, African nations have a roadmap for surveillance to eradicate cholera. Vaccination of 2 million people in the African continent took place in the first phase, which was carried out in 2018. The Global Vaccine Alliance (Gavi) conducted 5 major campaigns in Zambia, Uganda, Malawi, South Sudan, and Nigeria [20].

## CHOLERA ENDEMICITY IN INDIA

Studies carried out in India have shown that outbreaks of cholera are spreading among the increasing population. Socioeconomically deprived people, especially in rural areas and the urban poor, suffer the most from enteric diseases including cholera. These people reside in areas that also have an ecological and environmental risk of infections from cholera and other diseases.

In India, starting in 2000, the Indian Council of Medical Research (ICMR) led by N.K.G. gave impetus to studies on cholera. The first 2 studies in India were carried out by the National Institute of Cholera and Enteric Diseases (NICED), an ICMR institute. They found that cholera cases in India were more widespread and numerous than reported by the National Health Profile, who notified the WHO. A study lead by N.K.G. and his team identified cholera hotspots in India where the disease spread in India from 2010 to 2015.

## DETECTING *VIBRIO CHOLERAE* PREVALENCE AND IDENTIFYING ENDEMIC REGIONS

In October 2009, the WHO Strategic Advisory Group of Experts on Immunization (SAGE) formulated a criterion of endemicity. The group recommended that an area can be confirmed as endemic for cholera if there are reports of cholera cases in those areas for at least 3 of the past 5 years [21]. This method was to be implemented to identify areas endemic for cholera in India. However, because data sets of cholera cases were lacking, NICED used another scientific approach to identify endemicity.

The NICED Kolkata, in West Bengal, India, is a WHO Collaborating Center for Diarrhoeal Diseases Research and Training. The NICED, Kolkata receives approximately 1000 to 1500 strains of *V cholera* from several institutions in India and abroad annually. Between 1990 and 2007, NICED received more than 16 000 strains of *V cholera* from 24 different states of India for serotyping, biotyping, and phage typing. More than 7000 types have been incorporated in the phage-typing studies at NICED from 1990 to 2007 [22]. Cholera strains are classified by standardized methodology at NICED, which involves serotyping using polyvalent O1 and monospecific Inaba and Ogawa antisera, and a monoclonal antibody O139 (Difco, USA). The NICED use a standard form of universally adopted phage-typing methods [23, 24].

The disease has reoccurred in the following regions for 3 or more years including Andhra Pradesh, Delhi, Goa, Gujarat, Karnataka, Madhya Pradesh, Maharashtra, Punjab, Rajasthan, Tamil Nadu, and West Bengal, which were found to be endemic for the disease. The NICED studies have shown that many regions of India are endemic to cholera. Twenty-eight states in India provided strains and 11 states were found endemic for the disease. Maharashtra and West Bengal were found to have the maximal number of strains. Kerala and Sikkim were states added since 2004, which are now in the category of cholera-affected regions. The majority (96.5%) of the strains were the Ogawa serotype. Inaba contributed 3.5%. Basu and Mukherjee methods of phage typing indicated that type 27 was a predominant phage-type of cholera in India, followed by type 26 [22, 25, 26]. This definitive long-term study suggests that cholera was endemic over larger areas of India than was previously suggested. Cholera cases reported by the government to the WHO were significantly lower than the strains received for phage typing at NICED during 1996–2006. The study highlighted a lack of a more comprehensive and systematic surveillance system and supportive laboratory infrastructure. In a population-based study of diarrheal cases in the suburban Kolkata area, researchers found that cholera has been endemic with 2.2 cholera cases reported per 1000 cases of diarrhea [22, 27].

## CHOLERA SPREAD IN INDIA

To further track the actual number of cholera cases in India from 1997 to 2006, NICED carried out a systematic search of biomedical literature for data on cholera and cases reported from 1997 to 2006. The data were compared with the number reported to the WHO in the same period (1997–2007). India’s *National Health Profile* of 2006 also cited the number of cases reported by the government to the WHO [28]. The biomedical literature search indicated 68 outbreaks of cholera in 18 states. The NICED study showed that a total of more than 200 000 cases of cholera have been observed from 1996 to 2007. This was a significantly higher number than reported to the WHO, with only approximately 37 000 cases from India (Table 1). Overall, 21 states recorded cholera annually from 1996 to 2007. Ninety-one percent of all cases were reported from 4 states: West Bengal, Orissa, Assam, and Chhattisgarh [29]. Andaman Nicobar Islands were among the top 5 region territories with maximal numbers of cholera cases from 1996 to 2007. Although the study had its limitations, it demonstrated that the number of cases in India was underreported and that the methods being used for surveillance were inadequate.

**Table 1.** Delineating Cholera Spread in India^a^

Studies on Cholera	No. of States Affected Cholera/No. of States/Union Territories in the Study	No. of Patients Reported by the Study	Year of Surveillance Data Studied
Ali M, et al. [31]	24/36	27 615	2010–2015
Kanungo S, et al. [29]	21/25	222 038 (Only 37783 cases were recorded by existing surveillance system and reported to World Health Organization)	1996–2007

^a^The studies showed that the majority of cholera cases are underreported in India.

## IDENTIFYING CHOLERA HOTSPOTS

Interventions to eradicate cholera can be undertaken only by knowing which areas are hotspots of cholera. To identify current endemic and epidemic regions, mapping of cholera hotspots in India was carried out from 2010 to 2015. N.K.G. and a team comprising international collaborators, including John Hopkins University, analyzed the data of cholera outbreaks from India. Datasets of the Integrated Disease Surveillance Program (IDSP) was used to analyze the cholera outbreaks. The IDSP program was launched with the assistance of the World Bank in 2004 and was gradually integrated into the Government of India’s 5-year plans [30]. The IDSP identified disease outbreaks at the district level, and the source of information was used to identify cholera hotspots in India. The study involved the assembling of data of cholera disease outbreak regions as well as socioeconomic data from 2010 to 2015. The 2011 Census provided the socioeconomic factors and water and sanitation data in the areas that were affected by cholera.

## THE CHOLERA HOTSPOTS WERE IDENTIFIED USING SPATIAL ANALYSIS

The study found more than 27 000 cholera cases recorded from 2010 to 2015 (Table 1). All 28 states and 8 union territories (a total of 36 entities) were part of the mapping. Twenty-four states reported cases of cholera, whereas 13 endemic states were identified. Six hundred forty-one district-level states were included in the survey, 78 of which were hotspots. This was based on the number of cases that have been reported. However, upon further analysis of risk factors, 111 districts were deemed to be at risk of being hotspots for cholera. This increased risk was based on an assessment of socioeconomic parameters and several other factors that increase the propensity of cholera spread. Aspects that reduced the risk of cholera spread were associated with high literacy level, access to telecommunications, water and sanitation facilities, and other factors indicative of a healthy lifestyle. Those districts that were without proper drinking water and drainage, urban amenities, and health access were at higher risk for cholera transmission and outbreaks, and these were termed high-risk zones [31]. The study reinforced the notion that cholera is still underreported in large parts of India and further identified potential new hotspots. This study underscored the fact that policymakers should take note that cholera is still very prevalent in India and a national public health threat [31]. The areas most affected by cholera are mainly the rural areas that lack proper sanitation and health facilities.

## PREVENTION, PREPAREDNESS, AND EARLY WARNING: THE ROLES OF SURVEILLANCE SYSTEMS

Surveillance systems should be sensitive and reliable enough to collect all necessary data that can be utilized to benefit public health. For the surveillance system to be adequately robust, certain parameters need to be considered (Table 2). The data should be significant enough to establish the disease trends over time, that is, based on epidemiological patterns as well as accurately predict when future outbreaks may be highly likely.

**Table 2.** Surveillance Systems: Qualities

Surveillance No.	Attributes of Disease Surveillance System	Surveillance System Strengths
1	Indicator-based surveillance system	The laboratory system should be able to predict the longer term trends of diseases and detect the disease with sensitivity and specificity.
2	Notifiable disease surveillance system	The predicted trends should be monitored and detected in real time and recorded reported to concerned policymakers and the public in general.
3	Event-based surveillance	The surveillance system can detect all disease events irrespective of the location of occurrence, and all-time vigilance is important and provided by the program.
4	Rapid risk assessment Surveillance	The system is constantly capable of and able to assess a developing situation to provide reliable data sets for the public health professional for appropriate decision making.
5	Rapid response and when required permit funding for research in surveillance	The surveillance framework should have adequate funding and research infrastructure. The program managers in the system should have decision making capability and resources to carry analytical epidemiological studies during outbreaks and have the further mandate to carry out research to identify risk factors for outbreaks as well new diseases in the community.
6	An improved diagnostic that could help support improved cholera surveillance in India	Although the signs and symptoms of severe cholera can be easily identified in locations where it is prevalent, the only way to be sure is to identify the bacteria in a stool sample. Doctors in rural places might use rapid cholera dipstick tests to confirm a cholera diagnosis. Now, molecular technologies provide a quick confirmation of disease and thus reduces the number of people who die at the outset of a cholera outbreak and allows for earlier public health efforts to manage the disease. The Delivering Oral Vaccine Effectively (DOVE) program can be implemented in India.

^a^Major responsibility of a sensitive and robust surveillance system is to detect predict disease trends.

Cholera continues to be one of the major health concerns, especially in the Bay of Bengal region and other areas where it is endemic. Cholera outbreaks are a regular occurrence in these areas and have a propensity to appear seasonally. Reported cases do not reflect the actual extent of the disease in the Indian subcontinent. The surveillance system limitations are a major drawback, and, therefore, many are cases not reported. The majority of the cases that are registered are outbreaks only, because there has been no dedicated surveillance system in place.

Prediction about disease outbreaks becomes a valuable tool for public health officials. They will be able to anticipate the epidemics and take appropriate measures, such as stockpiling vaccines and drugs and creating training modules, so that healthcare workers are prepared for any eventuality.

Previous studies helped to identify the regions that require urgent intervention (ie, WASH) along with immunization. Control of cholera can be achieved by providing community water sanitation and hygiene, and WASH is one of the main interventions for the prevention of cholera in endemic communities. International guidelines, including WHO guidelines, are available to implement the WASH program in endemic areas, especially when implemented correctly at the community level. Nonetheless, contemporary evidence suggests that the majority of cases occur at the household level. Hence, a guideline might be more focused on the prevention of transmission from close contacts within a household [32, 33]. Thus, vaccination against cholera becomes of paramount importance and may be more sustainable than WASH interventions in some areas.

A robust and sensitive surveillance system can provide the impetus for decision makers to implement a sustainable vaccination program that can be implemented in endemic areas. We concluded that reliable epidemiological data are a crucial link for being prepared for a disease outbreak and, more importantly, to prevent outbreaks of cholera and other diseases [34]. Well planned immunization using oral cholera vaccine in endemic areas in addition to improvement in hygiene and the provision of portable drinking water can be worthwhile because it can help to control and eradicate the disease from endemic hotspots.

## WAY FORWARD

An expert group was formed in India and formulated a mechanism to gather new evidence to create a roadmap for cholera. The committee aims to address the issues related to the introduction of an OCV along with WASH in a systemic and time-bound manner such that it is sustainable to achieve positive results by the end of the program [35]. The experiences gained from the introduction of OCV in Vietnam and other countries demonstrate the effectiveness of the vaccine in controlling cholera. The way forward would be to provide a roadmap that empirically gives a clear direction of increased surveillance in cholera hotspots and detecting new cases and outbreaks. The action point from the policymaker’s perspective should be an integrated approach of research and development and socioeconomic development [36]. A surveillance system combined with a sustainable vaccination program that effectively delivers vaccinations to the most endemic areas of cholera is needed. We can refer to a John Hopkins University program, known as “Delivering Oral Vaccine Effectively” (DOVE), to learn how to implement an effective surveillance and vaccination program.

The focus of this project has been that vaccination is given to high-risk populations in cholera-endemic areas. The program has proven to be instrumental in preventing, treating, and controlling cholera in endemic areas. The program provides tools and resources to countries and public health organizations and agencies involved in cholera prevention and control. A strategic area of the program is providing evidence-based, decision-making information that can help in mitigating the threat of outbreaks in those areas where the program is implemented. StopCholera.org and the Stop Cholera Toolkit are a collection of practical tools and new resources created for the use of OCV. An integrated strategy was adopted using the practical tools provided to reduce cholera mortality and prevent new cases and outbreaks.

Developing nations have benefited from the DOVE program. This includes Bangladesh, Cameroon, Malawi, Nepal, Nigeria, South Sudan, Uganda, and Zambia. One of the highlights of the program is developing molecular and immune-detection techniques that the DOVE team developed for improved cholera surveillance in Bangladesh, Nepal, and several countries in Africa [37–40]. The work by Debes et al [38, 39] and Bénard et al [40] demonstrated that new technologies made it possible to develop rapid diagnostics techniques for cholera. Furthermore, the isolation and identification of new strains using genome sequencing enables rapid identification of hotspots with disease outbreaks [38–40]. The introduction of molecular diagnostic technologies along with the development of new effective surveillance tools coupled with vaccination is a game-changer for the prevention of cholera outbreaks in low-resource settings. Thus, the DOVE program demonstrates that resource allocation prioritization can lead to the creation of practical tools that effectively control cholera. Improved surveillance in conjunction with immunization programs needs to be implemented for the eradication and elimination of cholera in India.

## Notes

***Financial support.*** Delivering Oral Vaccine Effectively (DOVE) is supported by a grant from the Bill & Melinda Gates Foundation (OPP1053556) and is administered by the Johns Hopkins Bloomberg School of Public Health.

***Potential conflicts of interest*.** All authors: No reported conflicts of interest. All authors have submitted the ICMJE Form for Disclosure of Potential Conflicts of Interest.
